# Use of healthcare services preceding HIV diagnosis – missed opportunities for earlier diagnosis, Finland, 1996 to 2019

**DOI:** 10.2807/1560-7917.ES.2025.30.18.2400610

**Published:** 2025-05-08

**Authors:** Sanna Isosomppi, Mikaela Mutru, Jukka Ollgren, Henrikki Brummer-Korvenkontio, Kirsi Liitsola, Jussi Sutinen, Inka Aho, Pia Kivelä

**Affiliations:** 1 Epidemiological Operations Unit, City of Helsinki, Finland; 2 Department of Public Health, Finnish Institute for Health and Welfare, Helsinki, Finland; 3 HUS Inflammation Center, University of Helsinki and Helsinki University Hospital, Helsinki, Finland; * These authors contributed equally to this work and share first authorship

**Keywords:** HIV, Finland, Delayed diagnosis, Health Services

## Abstract

**Background:**

HIV testing based on indicator conditions is recommended to diagnose HIV earlier.

**Aim:**

Our aim was to assess opportunities for earlier diagnosis of HIV.

**Method:**

This is a retrospective study on people living with HIV (PLWH) included in the national HIV register. We collected data on public primary outpatient healthcare (PHC) (2011–2019), secondary and tertiary outpatient healthcare (STHC), and all inpatient care (1996–2019) from the Care Register for Health Care from the presumed acquisition, estimated by CD4^+^ T-cell count at diagnosis, until the diagnosis of HIV.

**Results:**

Of 907 PLWH diagnosed between 2011 and 2019, 522 (58%) had ≥ 1 healthcare contact at any level between HIV acquisition and > 30 days before diagnosis. At least one European Centre for Disease Prevention and Control (ECDC) indicator condition was recorded for 119 (23% of 522), and 112 (21%) were born in a high-prevalence country. In total, 384 of 907 (42%) had visited a PHC physician, and 58% of those with CD4^+^ T-cell count < 200 cells/μL at diagnosis. Of 2,082 PLWH diagnosed between 1996 and 2019, 869 (42%) had STHC outpatient contacts > 30 days before diagnosis, 18% with ≥ 1 ECDC indicator condition, and 367 (18%) had been hospitalised, 20% with ≥ 1 ECDC indicator condition. The most common ECDC indicator conditions > 30 days before diagnosis at all levels of healthcare were pneumonia, sexually transmitted infections, unexplained fever, herpes zoster, pregnancy and lymphadenopathy.

**Conclusion:**

We recommend enhancing indicator condition-based HIV testing by all healthcare providers, particularly for gonorrhoea, syphilis and, for persons younger than 50 years, also herpes zoster and lymphadenopathy.

**Key public health message**

**What did you want to address in this study and why?**

HIV causes a chronic infection that, if treated, does not reduce life expectancy and is not transmitted onward. Unfortunately, many are diagnosed late. Indicator conditions are diseases more common in people living with HIV (PLWH) because they are transmitted by the same route or more easily affect people with HIV-caused immunosuppression. We investigated how many could have been diagnosed earlier if tested when seeking healthcare for an indicator condition.

**What have we learnt from this study?**

More than half of PLWH had visited public healthcare for any reason before being diagnosed with HIV. The most common indicator conditions at those consultations were pneumonia, sexually transmitted infections, unexplained fever, herpes zoster (shingles) and unexplained swollen lymph nodes.

**What are the implications of your findings for public health?**

We recommend that people with indicator conditions should be tested for HIV. Some indicator conditions, such as sexually transmitted infections, can be a signal that the person is at risk of contracting HIV. In these cases, it is also important to provide information and other ways to prevent HIV.

## Introduction

Delayed diagnosis and thus treatment is a major driver of the HIV epidemic globally. Even in many high-income countries, the mean time from acquisition of HIV infection to its diagnosis is estimated to be around 3 years [1]. Timely antiretroviral (ART) treatment decreases risk of morbidity and mortality for the individual, and it effectively prevents onward transmission of HIV to other people [2,3].

Testing for HIV is recommended for persons diagnosed with HIV indicator diseases or conditions or those who have social or behavioural risk factors that are more common among people living with HIV (PLWH) [4,5]. Indicator condition-guided testing for HIV has been shown to be cost-effective if the suspected undiagnosed HIV prevalence in the target population is above 0.1% [6,7].

In high-income, low-prevalence settings, untargeted screening for HIV is not considered cost-effective [6,7]. In Finland, HIV screening is recommended in antenatal care, for babies born to women with HIV, and for blood, breast milk and organ donors. Currently, HIV testing is recommended for migrants coming from a high-prevalence (≥ 1%) country, and for everyone with indicator conditions, risk factors or symptoms indicative of HIV. Testing should also be offered at a person’s request [8,9].

The CD4^+^ T-cell count indicates the stage of HIV infection. Generally, for those who have not received long-term antiretroviral treatment, CD4^+^ T-cell count can be used as a surrogate for the time since acquisition of infection.

Healthcare contacts after acquisition of HIV infection are a possible testing opportunity that might enhance earlier diagnosis. An individual’s healthcare seeking behaviour, other medical conditions and access to healthcare, and a provider’s ability to follow guidelines affect this approach.

In 2019, an estimated 3,139 PLWH lived in Finland, corresponding to an HIV prevalence of 0.06% [10,11]. Approximately 6.6% of them were undiagnosed [10]. The rate of annual new diagnoses was 2.7 per 100,000 [12]. Treatment and care for HIV are provided free of charge for residents. All public healthcare is largely tax-funded and available for all residents in Finland.

This study aimed to assess opportunities for earlier diagnosis of HIV by analysing the quantity and diagnoses for public healthcare contacts of PLWH in Finland between presumed acquisition and diagnosis of HIV. Our objectives were (i) to report the total number of healthcare contacts preceding HIV diagnosis, stratified by type of visit and diagnosis, (ii) to determine how the number of healthcare contacts per persons in follow-up changed in the time preceding HIV diagnosis, stratified by type of visit and CD4^+^ T-cell count at diagnosis, and (iii) to report the total number and most frequent indicator conditions of HIV infection according to the Finnish and the European Centre for Disease Prevention and Control (ECDC) HIV testing guidelines. In addition, we examined how many PLWH who had contact with healthcare before HIV diagnosis had been born in a high-prevalence country warranting post-immigration opt-out screening.

## Methods

This was a registry-based retrospective cohort study. A unique personal identity code (PIC) is given to each person registered in Finland’s Population Information System [13]. We used PIC to link data from the national HIV register (FINHIV; previously named National HIV Health Care Quality Register), the Care Register for Health Care (Terveys-Hilmo, Health-Hilmo) and the Register of Primary Health Care Visits (AvoHilmo; a subset of Terveys-Hilmo).

### Data sources

The FINHIV was formed in 2020 at the Finnish Institute for Health and Welfare (THL). It is described in detail elsewhere [10]. The FINHIV was granted permanent status from 1 January 2023 [14]. Briefly, it combines data from all 21 public HIV clinics that treat PLWH in Finland with data from the National Infectious Disease Register (NIDR) that is maintained by THL and contains all notified HIV cases diagnosed and/or treated in Finland from 1 January 1984 onwards. Notification to NIDR is mandatory for physicians and for clinical laboratories [15]. From the FINHIV, we collected variables relevant for determining the time between HIV acquisition and diagnosis as well as key variables relevant to HIV infection, such as common co-infections: PIC, date and country of birth, sex, date of HIV diagnosis and whether diagnosis occurred before immigration, immigration date (if available), CD4^+^ T-cell count, HIV viral load, mode of transmission, and whether AIDS, hepatitis B or hepatitis C had been diagnosed.

The care registers on social and healthcare contacts (Hoitoilmoitusrekisterit, Hilmo) in Finland are maintained by THL [16]. They produce national statutory data on the availability and activities of healthcare for purposes of statistics, research, development and planning. Terveys-Hilmo, launched in 1994, contains data on all levels of inpatient care and outpatient visits to secondary and tertiary healthcare (STHC). Data on inpatient care using ICD-10 classification is available from 1996 for both public and private inpatient care, and for day surgery and public STHC outpatient contacts from 1998. Terveys-Hilmo contains data on service provider, client, date and type of appointment for outpatient care, date of admission and discharge for inpatient care, and diagnoses [17]. AvoHilmo data collection has covered all publicly provided primary healthcare (PHC) outpatient visits delivered in Finland since 2011 [18]. Providing preventive occupational healthcare is mandatory for employers in Finland. In addition, many employers provide access to medical care for their employees, mostly organised by private healthcare companies. Very few data on outpatient private healthcare are available in AvoHilmo for the study period as this data collection was about to begin in 2019. We collected the following information from Terveys-Hilmo and AvoHilmo: PIC, date of contact (arrival and discharge dates for inpatient care), type of contact (visit, inpatient care, remote contact), profession of contact (physician, nurse, dentist), whether outpatient contact was emergency or elective care, and all recorded diagnoses.

The data from the registers were linked using PICs at THL and pseudonymised before analysis.

### Study population

The study consisted of all PLWH in the FINHIV register with date of diagnosis between 1 January 2011 and 31 December 2019 for PHC contacts, and between 1 January 1996 and 31 December 2019 for inpatient care and STHC contacts. Exclusion criteria were invalid PIC, diagnosis before immigration, CD4^+^ T-cell count not available within 180 days of diagnosis and HIV viral load < 200 copies/mL within 90 days of recorded diagnosis, which is most probably due to ongoing HIV treatment i.e. a diagnosis that is not recent.

Time from presumed acquisition of HIV infection to diagnosis was based on a study by Lodi et al. [19]. It was defined by CD4^+^ T-cell count as follows: ≥ 500 cells/μL indicates diagnosis 6 months after HIV acquisition, 350–499 cells/μL 1 year, 200–349 cells/μL 3 years and < 200 cells/μL 5 years. For each person, we collected the healthcare contacts from the presumed HIV acquisition date up to the day before the HIV diagnosis date, which is most often recorded as the day when the positive HIV test was taken.

When determining the number of healthcare contacts before HIV diagnosis per persons in follow-up, the beginning of the follow-up was the presumed date of HIV acquisition except for those who moved to Finland in the time between presumed acquisition and diagnosis of HIV. For them, the beginning of the follow-up was defined as the earliest recorded date available from the registries we used for this study. This was the date when a residence permit was granted, when the first municipality of residence in Finland recorded, or when the first healthcare contact was recorded.

We used data from Terveys-Hilmo for inpatient care from 1 January 1996, for STHC outpatient contacts from 1 January 1998, and data for PHC contacts (from AvoHilmo) from 1 January 2011 until 31 December 2019. We further divided PHC contacts into visits to physicians, dentists and nurses. For contacts to nurses, data were collected only from actual visits, as we considered it more likely that the nurse might in those circumstances have consulted a physician, and thus an HIV test had been offered. Data on visits to dental nurses or nurses working in substance use services were not collected. The PHC contacts included outpatient visits only; remote contacts such as phone calls, text messages or other electronic messages were excluded. The STHC outpatient contacts may include some phone calls that substituted for a follow-up visit, and some visits to professionals other than physicians because they could not be differentiated from the data.

Day surgery was included in STHC outpatient contacts. A period of inpatient care was recorded by the discharge date as that was the last opportunity of diagnosing HIV. We defined adjacent periods of inpatient care recorded by different specialties as a single period, and included emergency care visits leading to hospitalisation in the inpatient period. For outpatient care, five or more visits to the same specialty on consecutive days without breaks were considered as one outpatient treatment period ; fewer than five such visits were considered as separate visits. In 2024, more than 98% of diagnoses recorded by PHC physicians and dentists were recorded according to the ICD-10 classification but at the time of study, approximately 10% of diagnoses at PHC physician contacts were still recorded according to ICPC-2 classification [20]. Our main focus were diagnoses recorded by physicians. Because the ICPC-2 diagnoses were also recorded by other healthcare personnel and were rarely used by physicians, we only examined ICD-10 diagnoses.

We defined healthcare contacts more than 30 days before diagnosis as possible missed opportunities and assessed them separately for the presence of indicator conditions. We used the Finnish HIV testing recommendation 2010 and the broader 2018 ECDC public health guidance on HIV and hepatitis B and C testing in the European Union and European Economic Area (EU/EEA) to classify the indicator conditions according to ICD-10 codes, and all ICD-10 diagnoses were also classified into categories. A list of the ICD-10 diagnoses assigned to each category or indicator can be found in the Supplement [4,8].

### Statistical analysis

The relevant characteristics of the study population are reported separately for the analysed time periods, 2011 to 2019 for analysis of PHC contacts and 1996 to 2019 for inpatient and STHC outpatient contacts, as absolute numbers and as percentages of the total study population. The proportion of the study population with healthcare contacts is reported in total and per characteristic. We analysed whether the proportion of PLWH with healthcare contacts differed by characteristic (e.g. between men and women). For this we used chi-squared test for categorical variables and Mann–Whittney U test for age at diagnosis, as it was not normally distributed. We used Stata version 17 (StataCorp LLC) for analysis. The number of healthcare contacts are reported in total and as median and interquartile range (IQR). The percentage of STHC outpatient contacts due to emergency care is reported. Main ICD-10 diagnosis recorded for each type of contact (PHC physician visit, inpatient care and STHC outpatient contacts) is reported by category. The number of healthcare contacts per persons in follow-up was determined for each month preceding the date of HIV diagnosis and is presented graphically for each type of healthcare contact by different categories of CD4^+^ T-cell count at diagnosis (≥ 500 cells/μL, 350–499 cells/μL, 200–349 cells/μL and < 200 cells/μL). Physician contacts, the proportion of PLWH with recorded indicator diagnoses, and most common indicator diagnoses more than 30 days before HIV diagnosis are reported by type of contact. We analysed the same variables for time less than 30 days before diagnosis for comparison. For the period 2011 to 2019, when data from both PHC and STHC were available, we analysed the proportion with contacts or recorded indicator diagnoses at any level of healthcare. The proportion of PLWH who were born in high-prevalence countries of those who had healthcare contacts is reported.

## Results

**Figure 1:**
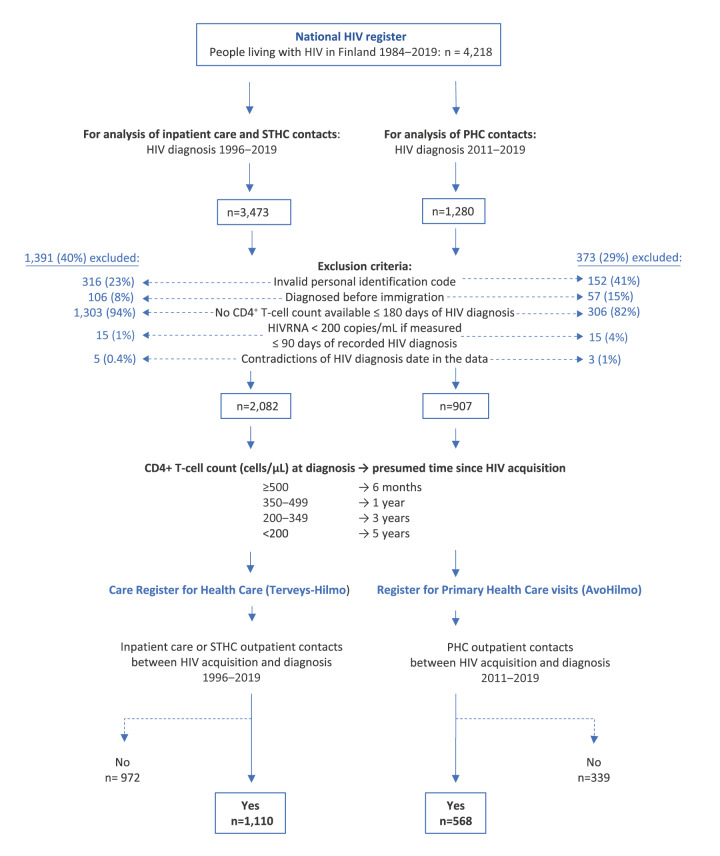
Flow chart of collection and processing of data for the study, Finland, 1996–2019 (n = 4,218)

STHC: secondary and tertiary healthcare; PHC: primary healthcare.

The data collection and processing flow chart is shown in Figure 1.

Data on people living with HIV were collected from the national HIV register for the study. Exclusion criteria and people excluded based on them are reported out of all excluded, i.e. a person could have multiple reasons for exclusion. For the people included in the study, the time from presumed HIV acquisition to diagnosis was determined based on the CD4^+^ T-cell count recorded at diagnosis in the national HIV register. Healthcare contacts from Care Register for Health Care and Register for Primary Health Care Contacts were collected for the time between presumed acquisition and diagnosis of HIV.

### Public primary healthcare contacts 2011–2019

Of those diagnosed with HIV for whom data on primary healthcare were available (2011–2019), 907 PLWH were included in the study, and 568 (63%) had a total of 3,833 PHC contacts between presumed acquisition and diagnosis of HIV: 1,828 visits to physicians (median = 2; IQR: 1–5 for those who visited), 1,283 to nurses (median = 2; IQR: 1–4) and 722 to dentists (median = 2; IQR: 1–4). The characteristics of the PLWH diagnosed in the period 2011 to 2019 are presented in Table 1.

**Table 1:** Characteristics of the study subpopulation and whether they had recorded primary healthcare contacts between HIV acquisition and diagnosis, Finland, HIV diagnosis 2011–2019 (n = 907)

	Population		Proportion with primary healthcare contacts		p value^a^
	n	% of all	n	% of row	
All	907	100.0	568	63.6	NA
Sex					
Female	239	26.4	154	64.4	0.500
Male	668	73.6	414	62.0	
Country of birth					
Finland	573	63.2	371	64.7	0.253
High-prevalence^b^	255	28.1	151	59.2	
Other	76	8.4	45	59.2	
Unknown	3	0.3	1	33.3	
Mode of transmission					
Heterosexual	508	56.0	329	64.8	0.018
MSM	314	34.6	179	57.0	
IDU	20	2.2	14	70.0	
Other	8	0.9	2	25.0	
Unknown	57	6.3	44	77.2	
Year of diagnosis					
2011–2013	319	35.2	191	59.9	0.412
2014–2016	333	36.7	211	63.4	
2017–2019	255	28.1	166	65.1	
Median age at diagnosis (IQR)	40.0 (31.6–51.6)		42.1 (32.3–53.6)		< 0.001
CD4^+^ T-cell count at diagnosis^c^					
≥ 500 cells/μL	255	28.1	113	44.3	< 0.001
350–499 cells/μL	156	17.2	79	50.6	
200–349 cells/μL	187	20.6	136	72.7	
< 200 cells/μL	309	34.1	240	77.7	
AIDS^d^	164	18.1	120	73.2	0.002
Hepatitis B S-antigen					
Positive	17	1.9	9	52.9	0.438
Negative	735	81.0	457	62.2	
Unknown	155	17.1	102	65.8	
Hepatitis C antibodies					
Positive	54	6.0	36	66.7	0.459
Negative	695	76.6	428	61.6	
Unknown	158	17.4	104	65.8	

IDU: injection drug use; IQR: interquartile range; MSM: men who have sex with men; NA: not applicable.

^a^ Chi-squared test for difference by characteristic in proportion of those with contacts to primary healthcare and those with no such contacts. Mann-Whittney U test was used for median age at diagnosis.

^b^ Prevalence > 1% [33].

^c^ Measured within 180 days after HIV diagnosis.

^d^ Diagnosed with an AIDS indicator condition: does not include people with CD4^+^ T-cell count < 200/μL without concurrent AIDS-defining illness.

### Inpatient and public secondary and tertiary healthcare outpatient contacts 1996–2019

For inpatient and STHC outpatient contacts, 1,110 of 2,082 PLWH diagnosed between 1996 and 2019 (53%) had at least one healthcare contact between presumed acquisition and diagnosis of HIV (Table 2).

**Table 2:** Characteristics of the study subpopulation and whether they had secondary and tertiary healthcare outpatient care or inpatient care between HIV acquisition and diagnosis, Finland, HIV diagnosis 1996–2019 (n = 2,082)

	Population		Proportion with healthcare contacts^a^		p value^b^
	n	% of all	n	% of row	
All	2,082	100.0	1,110	53.3	NA
Sex					
Female	559	26.8	272	48.7	0.010
Male	1,523	73.2	838	55.0	
Country of birth					
Finland	1,485	71.3	866	58.3	< 0.001
High-prevalence^c^	471	22.6	183	38.9	
Other	123	5.9	60	48.8	
Unknown	3	0.1	1	33.3	
Mode of transmission					
Heterosexual	995	47.8	513	51.6	< 0.001
MSM	720	34.6	365	50.7	
IDU	247	11.9	158	64.0	
Other	20	1.0	6	30.0	
Unknown	100	4.8	68	68.0	
Year of diagnosis					
1996–2001	278	13.4	138	49.6	0.093
2002–2007	540	25.9	270	50.0	
2008–2013	675	32.4	376	55.7	
2014–2019	589	28.3	326	55.3	
Median age at diagnosis (IQR)	37.9 (30.2–47.3)		40.4 (32.2–50.8)		< 0.001
CD4^+^ T-cell count at diagnosis^d^					
≥ 500 cells/μL	667	32.0	248	37.2	< 0.001
350–499 cells/μL	414	19.9	196	47.3	
200–349 cells/μL	418	20.1	237	56.7	
< 200 cells/μL	583	28.0	429	73.6	
AIDS^e^	368	17.7	281	76.4	< 0.001
Hepatitis B S-antigen					
Positive	43	2.1	23	53.5	0.837
Negative	1,609	77.3	835	51.9	
Unknown	430	20.7	252	58.6	
Hepatitis C antibodies					
Positive	229	11.0	144	62.9	< 0.001
Negative	1,422	68.3	714	50.2	
Unknown	431	20.7	252	58.5	

IDU: injection drug use; MSM: men who have sex with men; NA: not applicable; STHC: secondary and tertiary healthcare.

^a^ Includes public STHC outpatient contacts and any level of inpatient care.

^b^ Chi-squared test for difference by characteristic in proportion of those with contacts to healthcare and those with no contacts. Mann-Whittney U test was used for median age at diagnosis.

^c^ Prevalence > 1% [33].

^d^ Measured within 180 days after HIV diagnosis.

^e^ Diagnosed with an AIDS indicator disease: does not include people with CD4^+^-T cell count < 200/μL without concurrent AIDS-defining illness.

The main diagnoses are shown by category in Table 3. Approximately 23% of the outpatient contacts were in emergency care. Alcohol and substance use, infections, trauma, and ICD10 R-codes for symptoms, signs and abnormal findings were more common diagnosis categories in emergency care than in elective care. 

**Table 3:** Physician contacts between presumed HIV acquisition and diagnosis by type of contact to primary healthcare, secondary and tertiary healthcare or inpatient care. Finland, 1996–2019 (n = 2,082)

Type of contact	PHC physician		STHC outpatient		Inpatient (PHC/STHC)	
Year of diagnosis	2011–2019 (n = 907)		1998–2019 (n = 2,075)		1996–2019 (n = 2,082)	
	n	%	n	%	n	%
Persons with contact	455	50.2	1,010	48.7	556	26.7
Number of contacts	1,828		6,108		1,100	
Median of those with contacts (IQR)	2 (1‒5)		3 (1‒7)		1 (1‒2)	
Median of all diagnosed (IQR)	1 (0‒2)		0 (0‒3)		0 (0‒1)	
ICD-10 code available for visit	1,144	62.6	5,454	89.3	1,100	100.0
**Diagnosis category^a^**						
Infection	321	28.1	510	9.4	264	24.0
Malignancy and tumours	16	1.4	250	4.6	41	3.7
Alcohol or substance use and poisoning	6	0.5	290	5.3	129	11.7
Psychiatry	38	3.3	777	14.2	86	7.8
Neurology	17	1.5	197	3.6	21	1.9
Eyes, ears, throat, nose, mouth, teeth	59	5.2	499	9.1	28	2.5
Heart and lungs	69	6.0	278	5.1	73	6.6
Gastroenterology and urogenital diseases	70	6.1	501	9.2	128	11.6
Endocrinology	21	1.8	56	1.0	14	1.3
Trauma	77	6.7	420	7.7	69	6.3
Rheumatology	8	0.7	70	1.3	3	0.3
Bones and soft tissues (excluding trauma)	138	12.1	256	4.7	36	3.3
Haematology	9	0.8	124	2.3	75	6.8
Skin	66	5.8	261	4.8	15	1.4
Pregnancy-related	2	0.2	48	0.9	16	1.5
Symptoms (R-codes]	154	13.5	470	8.6	89	8.1
Z-codes and others	73	6.4	447	8.2	13	1.2

IQR: interquartile range; PHC: primary healthcare; STHC: secondary and tertiary healthcare.

^a^ Diagnosis categories and the ICD-10 diagnoses they include are listed in the Supplement. Only the main diagnosis was analysed. Proportion reported of all visits for which a diagnosis was available.

### The number of healthcare contacts before HIV diagnosis per persons in follow-up

**Figure 2:**
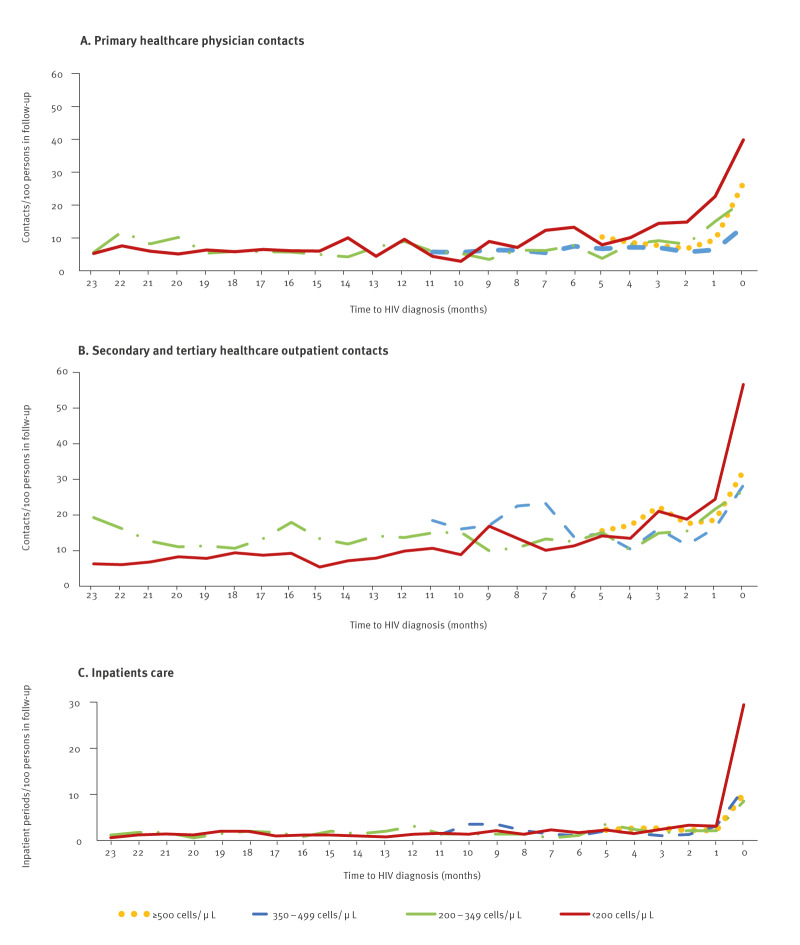
Frequency of healthcare contacts before HIV diagnosis, by time and CD4^+^ T-cell count at diagnosis, Finland, 1996–2019 (n = 1,971)

The total follow-up time from presumed acquisition of HIV or immigration to HIV diagnosis was 4,114 years for the period 1996 to 2019 (median = 1 year), 3,997 years for the period 1998 to 2019 (median = 1 year) and 1,584 years for the period 2011 to 2019 (median = 1 year). The number of all healthcare contacts per persons in follow-up increased rapidly during the month before HIV diagnosis, but a gradually rising trend in both PHC physician contacts and STHC outpatient contacts was seen in 6–12 months before diagnosis for those with CD4^+^ T-cell counts < 200 cells/μL (Figure 2).

### Contacts more than 30 days before HIV diagnosis and recorded indicator conditions

Contacts to healthcare more than 30 days before HIV diagnosis and recorded indicator conditions are presented in Table 4. The proportion of individuals with such contacts increased with age at all levels of healthcare: for PHC physician contacts, the proportion was 37% among those diagnosed at 20–40 years of age and 68% among those diagnosed at an age older than 60 years. For the same age groups, the proportions were, respectively, 35% and 66% for STHC outpatient contacts and 14% and 33% for inpatient care. Of those with CD4^+^ T-cell count < 200 cells/μL at diagnosis, 58% had visited a PHC physician, 57% had had STHC outpatient contacts and 28% had been hospitalised more than 30 days before diagnosis.

**Table 4:** Physician contacts and indicator diagnoses more than 30 days before HIV diagnosis, by type of contact to primary healthcare, secondary and tertiary healthcare or inpatient care, Finland, 1996–2019 (n = 869)

Type of contact	PHC physician 2011–2019		STHC outpatient 1998–2019			Inpatient care (PHC/STHC) 1996–2019		
	n	%		n	%		n	%
Persons with contacts	**384**	42.3^a^		**869**	41.9^a^		**367**	17.6^a^
With ≥ 1 ECDC indicator^b^	56	14.6		154	17.7		75	20.4
With ≥ 1 Finnish testing criterion^b^	24	6.3		104	12.0		46	12.5
Born in a high-prevalence country^c^	92	24.0		126	14.5		39	10.6
ECDC indicator and/or high-prevalence country of birth^b,c^	138	35.9		253	29.1		106	28.9
Number of contacts	**1,572**			**5,373**			**767**	
ICD10 code available for visit	986	62.7		4,774	88.9		767	100
With ECDC indicator^b^	87	8.8		394	8.3		106	13.8
With Finnish testing criterion^b^	34	3.4		290	6.1		69	9.0
Born in a high-prevalence country^c^	334	21.2		510	9.5		53	6.9
**Most common ECDC indicators^d^**	**n**	**%**		**n**	**%**		**n**	**%**
Herpes zoster	19	4.9	Pneumonia	18	2.1	Pneumonia	24	6.5
Pneumonia	11	2.9	Other STI^e^	17	2.0	Hepatitis B or C	19	5.2
Fever, unexplained	10	2.6	Pregnancy	17	2.0	Pregnancy	10	2.7
Lymphadenopathy	4	1.0	Syphilis	16	1.8	Fever, unexplained	9	2.5
Candidiasis (excl. genital candidiasis)	3	0.8	Chlamydia	13	1.5	Lymphadenopathy	6	1.6
Other STI^e^	3	0.8	Gonorrhoea	12	1.4	Leukopenia/ thrombopenia	3	0.8
Pregnancy	3	0.8	Fever, unexplained	11	1.3	Herpes zoster	3	0.8
			Lymphadenopathy	11	1.3	Chronic renal impairment	3	0.8
			Herpes zoster	10	1.2			
			Mononeuritis	10	1.2			

ECDC: European Centre for Disease Prevention and Control; PHC: primary healthcare; STHC: secondary and tertiary healthcare; STI: sexually transmitted infection.

^a^ % of diagnosed.

^b^ A list of ICD-10 diagnoses assigned to an ECDC indicator and the Finnish testing criteria can be found in the Supplement.

^c^ Prevalence > 1% [33].

^d^ Terveys-Hilmo records one main diagnosis + two additional (optional). Here all diagnoses are counted once per person, and the percentage is based on all persons who had contacts. Ten most common indicator diagnoses are reported per type of contact if they were recorded for at least three people.

^e^ Other STIs include for example genital herpes simplex virus and condyloma, but not chlamydia, gonorrhoea or syphilis.

Analysing contacts at all levels of healthcare for those diagnosed between 2011 and 2019, 674 of 907 (74%) had at least one recorded healthcare contact between HIV acquisition and diagnosis, and 522 (58%) had at least one contact more than 30 days before diagnosis. At least one ECDC indicator condition more than 30 days before HIV diagnosis was recorded for 119 (23% of those with contacts and 13% of all diagnosed) and 72 by the Finnish criteria (13% and 8%, respectively). The proportion of PLWH who had an ECDC indicator recorded more than 30 days before HIV diagnosis increased from 10% in 2011 to 2013 to 18% in 2017 to 2019. For STHC outpatient and inpatient contacts, the proportion of PLWH with an indicator recorded at more than 30 days before diagnosis increased from 8% in 2011 to 2013 to 13% in 2017 to 2019, while the proportion of PLWH who had an indicator any time before diagnosis recorded in STHC outpatient or any inpatient care remained in the same range (32–36%) during the whole period 2011 to 2019.

People born in a high-prevalence country constituted 28% (n = 255) of the 907 PLWH diagnosed between 2011 and 2019, and 112 (44%) of them visited public healthcare or were hospitalised more than 30 days before diagnosis. They constituted 21% of those diagnosed between 2011 and 2019 who visited public healthcare or were hospitalised more than 30 days before HIV diagnosis, and 209 (40%) were either born in a high-prevalence country and/or had an ECDC indicator diagnosis. There were no significant differences by country of birth in the proportion of PLWH with an ECDC indicator; 22 of 112 people (20%) born in high-prevalence countries who visited public healthcare more than 30 days before diagnosis had an ECDC indicator diagnosis recorded.

### Indicator conditions less than 30 days before HIV diagnosis

Of those diagnosed between 2011 and 2019, 371 (41%) visited public healthcare or were hospitalised 0–30 days before HIV diagnosis: 124 (33%) of them had an ECDC indicator diagnosis recorded, and 67 (18%) fit the Finnish testing criteria. In the 30 days preceding HIV diagnosis, the most common ECDC indicator conditions recorded at all levels of healthcare were pneumonia, unexplained fever, lymphadenopathy, hepatitis B or C, pregnancy and leukopenia/thrombopenia.

## Discussion

More than half of the PLWH had a contact to any level of public healthcare after HIV acquisition but more than 30 days before diagnosis. During that time, 23% had at least one ECDC indicator condition recorded, and 13% fit the Finnish testing criteria. The most common indicator conditions were pneumonia, sexually transmitted infections (STIs), herpes zoster, pregnancy, hepatitis B or C, unexplained fever and lymphadenopathy.

This study is based on national databases that cover all levels of public healthcare and private inpatient care and can be linked effectively by personal identity codes. To our knowledge, most previous studies from other countries are either regional or from single centres [21-32]. Data are often collected by questionnaires, which have an inherent recall bias [17-19,33]. There are some studies based on national registers but to our knowledge, few cover all levels of healthcare and include data on missed indicator conditions by ICD-10 codes [34,35].

In our study 42% of PLWH diagnosed between 2011 and 2019 had visited a PHC physician after HIV acquisition but more than a month before HIV diagnosis. In a Finnish population study where a random sample of the population were invited to participate in a survey, 34% of men and 42% of women reported visiting a public PHC physician in the 12 months before the survey [36]. The proportions increased with age similarly to our study. Considering the longer follow-up time for our study population (mean: 1.8 years), the proportions are comparable. In some other European studies, 71–93% reported a PHC physician contact in the years (1–5 years depending on the study) before HIV diagnosis [21,22,34,37]. The difference can probably be explained by the role of occupational healthcare in Finland, for which data were not available for our study: in addition to mandatory preventive occupational healthcare, many employers provide voluntary medical care to their employees, mostly contracted to private healthcare companies. For the Finnish population in 2011 it was estimated that public PHC accounted for 34%, STHC for 21%, occupational healthcare for 26% and private healthcare for 16% of physician visits [36]. In the survey to the general population in Finland, 13% reported being hospitalised in the last 12 months [36]. In our study, the proportion with inpatient care more than 30 days before diagnosis was higher, although the follow-up time was also longer: 18%, with a mean follow-up time of 2.1 years.

Psychiatric diagnoses were recorded for 14% of STHC outpatient contacts, largely explained by the treatment of some chronic disorders that require frequent contacts. Alcohol or substance use and intoxication were the main diagnoses for 5% of STHC outpatient contacts and 12% of inpatient care. These contacts were mostly from 1996 to 2005, coinciding with the HIV epidemic among people who inject drugs in the capital region in Finland: between 1996 and 2000, the proportion of STHC contacts due to these diagnoses was 38%, but it was less than 1% in the period 2011 to 2015. In our study, approximately two thirds of PLWH with transmission through injection drug use had STHC contacts or inpatient care before HIV diagnosis, compared with half of PLWH with transmission through sex (either heterosexual or sex between men). This suggests that enhanced testing in hospitals is more likely to increase early diagnoses among PLWH with injection drug use than among, for example, men who have sex with men.

Indicator condition-based testing has been promoted by the World Health Organization and ECDC since the early 2000s [5]. In our study, at least one ECDC indicator diagnosis was recorded more than 30 days before HIV diagnosis for 13% of the PLWH diagnosed between 2011 and 2019. The proportion increased towards the end of the decade which may be partly explained because a longer period of PHC data was available for those diagnosed in the later years. However, the proportion of PLWH with an indicator recorded at STHC or inpatient contacts more than 30 days before diagnosis also increased, while the proportion with an indicator recorded at any point before the diagnosis remained in the same range, suggesting that fewer with an indicator may have been tested in the latter half of the 2010s. If everyone with an ECDC indicator diagnosis had been tested for HIV, public healthcare could potentially have identified earlier up to one in six of those diagnosed between 2011 and 2019, and up to one in four of those who had public healthcare contacts. The Finnish testing recommendation from 2010 has more limited testing criteria, but 14% of those with healthcare contacts and 8% of all diagnosed fit those criteria as well. In other European studies, ECDC indicators before HIV diagnosis have been reported for 26–59% of PLWH, with subsequent HIV testing varying in the range 5–53% [22,25,26,28,31,32,34]. Creating reminders for clinicians in electronic health record programmes might enhance provider-initiated opt-out testing [38].

We compared the most common indicators in our data more than 30 days before HIV diagnosis with the number of diagnoses in the general population (THL open data [39-41]). Based on the comparison, the most impactful indicators to prompt testing, those with a prevalence of undiagnosed HIV in probably more than one in 1,000 healthcare contacts, would be syphilis, gonorrhoea and, in persons younger than 50 years, also herpes zoster and lymphadenopathy. In addition, STIs and hepatitis B and C are indicators of past or ongoing transmission risk and therefore would warrant opt-out HIV testing and assessing need for pre-exposure prophylaxis. Our data recorded 19 persons with pregnancy-related ICD-10 codes more than 30 days before diagnosis. Most of these were for spontaneous or induced abortions, or pregnancy-related symptoms. For persons planning to deliver, universal opt-out screening during pregnancy has been in place since 1997. Despite the policy of universal opt-out screening, some deliveries were recorded between HIV acquisition and diagnosis. They could be due to failing to offer or declining the test, or HIV acquisition after the test.

Of PLWH born in high-prevalence countries, 44% had contacts to healthcare > 30 days before diagnosis as HIV-infected. This was because post-immigration screening was either not offered, delayed or declined, or because transmission occurred after immigration. We did not have data on whether the positive tests were part of screening, nor on negative HIV tests. In the years 2015 and 2016, the coverage of infectious disease screening among eligible asylum seekers in Finland was estimated at 60–70%, and the average delay for blood tests from arrival was 91 days [42]. Opt-out screening for all migrants from high-prevalence countries is recommended but not systematically offered because information on recently arrived migrants except refugees and asylum seekers is not available to the healthcare authorities. The proportion of post-immigration transmission has been estimated at 19–40% in some European countries [43-45]. If HIV testing had been offered at any healthcare contact, 44% of PLWH born in high-prevalence countries could have been diagnosed earlier. Acceptance rate of opt-in HIV testing as a part of study of migrants in Finland was 92% when the study was conducted in the participants’ native language and pre-test counselling was optimised [46]. However, in 2022, up to 3.6% of the Finnish population was born in high-prevalence countries, and in some studies offering repeat testing based on only country of birth has been perceived as stigmatising [47-49]. We recommend improving coverage of post-immigration screening, and for those tested negative at arrival, enhancing primary prevention and offering an HIV test based on individual risk factors.

To control the HIV epidemic in Finland, universal access to healthcare should be ensured for all, regardless of the residence status, and low-threshold healthcare services and point-of-care HIV testing should be provided for sub-populations, such as people who use injection drugs and migrants who are less likely to use regular healthcare services. When taking our findings into practice, we also need to consider the effects of the changes in the healthcare system such as task-shifting from physicians to other healthcare professionals, especially in PHC, and the increasing proportion of healthcare contacts made through digital channels.

This study was based on register data that was primarily collected for surveillance and patient care. There might be some variations in the completeness of data over time and by reporting organisations. We did not have access to medical records for checking or adding any missing data. Mode of transmission of HIV might have been misreported or unreported due to stigma. We did not have data on private outpatient or occupational healthcare. Remote contacts and visits on the day of HIV diagnosis were not included. ICD10-codes were unavailable for a larger proportion of PHC contacts than STHC contacts. A third of PHC physician visits lacked an ICD10-coded diagnosis. These were excluded from analysis of indicator conditions. Hence, the proportion of PLWH who contacted healthcare because of an indicator condition is likely to be underestimated in our study. We had no data on why or where the positive HIV test was taken. Only 33% of those who visited public healthcare within 30 days before diagnosis had a recorded ECDC indicator condition. Some may have been tested because of indicator conditions recorded during contacts for which we did not have data. As an important limitation, testing based on behavioural and lifestyle factors, of which the healthcare provider might have been aware, cannot be assessed in this study.

The actual date of immigration was not available from our data sources, and it is likely to have been earlier than our follow-up start date. Thus, the duration of the follow-up period for some migrants is probably an underestimation. For the 111 foreign-born PLWH diagnosed between 1996 and 2019 who had no recorded healthcare contacts, we did not know the date of immigration and could not determine for how long they had been in the country before HIV diagnosis. In addition, many immigrants are given a temporary PIC after arrival before receiving a permanent one. Unlike permanent PICs, these are organisation-specific, and data linkage between them and the permanent PIC possibly issued later is incomplete. Healthcare contacts of immigrants that were recorded under a temporary PIC were not available for the study.

A temporary decline in CD4^+^ T-cell count may occur during primary infection. There is documented variety in the rate of CD4^+^ T-decline [50,51]. Data on possible negative HIV test results was not available. It is possible that some of the study population may have been infected later or earlier than we assumed. We excluded 15 PLWH who had HIV RNA < 200 copies/mL within 90 days of recorded diagnosis because it was likely that they had received ART earlier and the diagnosis was not recent. Some of them may have been recently diagnosed and treated with integrase inhibitors, which reportedly may lower the number of viral copies to undetectable in weeks [52].

## Conclusions

Improving provider-initiated testing based on the ECDC indicators, particularly syphilis, gonorrhoea and, for persons under 50 years, herpes zoster and lymphadenopathy, could enhance earlier diagnosis and treatment for the PLWH in Finland. People born in high-prevalence countries visited healthcare and were diagnosed with indicator conditions before HIV diagnosis. This indicates that the current post-immigration screening does not reach all eligible migrants. Besides screening with accompanying information on preventive measures, HIV testing should be offered at diagnosis of an indicator condition or based on individual risk factors. A quarter of the PLWH diagnosed in the period 2011 to 2019 did not visit a public healthcare physician before HIV diagnosis. They may have had contact to private or occupational healthcare however, so it is important to ensure that recommendations regarding HIV testing at diagnosis of an indicator condition are also followed by physicians outside public healthcare. Diagnosis of indicators of past or present transmission risk such hepatitis B or C or STIs provide opportunities to diagnose HIV earlier and to enhance prevention. Combining HIV testing based on indicator conditions and based on behavioural and lifestyle risk is likely to be the most cost-effective strategy for diagnosing HIV earlier in low-prevalence settings.

## Data Availability

The data that support the findings of this study are available on request for scientific research in anonymised form from Data Permit Authority Findata (https://www.findata.fi/en/services/data-requests/). The data are not publicly available due to privacy or ethical restrictions.

## Supplementary Data

121000 Supplement
