# Supplementary material for: Use of healthcare services preceding HIV diagnosis – missed opportunities for earlier diagnosis, Finland, 1996 to 2019
Source: Euro Surveill. 2025 May 8;30(18):2400610. doi: 10.2807/1560-7917.ES.2025.30.18.2400610 (PMC12066979; doi:10.2807/1560-7917.ES.2025.30.18.2400610)
Supplement: Supplement [file 24-00610_MUTRU_Supplement.pdf]

Supplementary material

This supplementary material is hosted by *Eurosurveillance* as supporting information alongside the article Use of health care services preceding HIV diagnosis in Finland – missed opportunities for earlier diagnosis, on behalf of the authors, who remain responsible for the accuracy and appropriateness of the content. The same standards for ethics, copyright, attributions and permissions as for the article apply. Supplements are not edited by *Eurosurveillance* and the journal is not responsible for the maintenance of any links or email addresses provided therein.

Table S1. Disease categories and indicator conditions by ICD10 coding

**Disease category**	**ICD10 included**		
Infection	A, B, G0, H60.0–H60.3, H66–H67, H68.0, H70.0, I33, I38–I39, J00–06, J09–J18, J20–J22, J36, J39.0, J39.1, K11.2, K11.3, K12.2, L00–L09, M00–M01, M86, N10–N12, N30, N41, N45, N61, N70–N77		
Malignancy and tumours	C, D0–D44, D48, N87		
Alcohol and substance use and poisoning	F1, T4, T51, T52, Y90–91		
Psychiatry	F (excluding F1)		
Neurology	G1–G9		
Eyes, ears, throat, nose, mouth, teeth	H0–H5, H60.4–H65, H68.1–H69, H70.1–H95, J30–J35, J37–J38, J39.2–J39.9, K0–K11.1, K11.4–K12.1, K13–K14		
Heart and lungs	I0–I32, I34–I37, I40–I9, J4–J9		
Gastroenterology and urogenital diseases	K2–K9, N0, N13–N2, N31–N40, N42–N44, N46–N50, N62–N64, N80–86, N91–98		
Endocrinology	E		
Trauma	S, T (excluding T4, T51 and T52), V, W, X, Y94–Y96		
Rheumatology	M02–M14, M3, M45–M46		
Bones and soft tissues	M15–M25, M40–M44, M47–M48, M50–M54, M6–M85, M87–M9		
Haematology	D45–D47, D50–D89		
Skin	L1–L9		
Pregnancy-related	O, Z34, Z35		
Symptoms, signs and abnormal findings	R		
Z-codes and others	Z (excluding Z34 and Z35), P, Q		

Indicator condition	ICD10 included	ECDC indicator[a]	Finnish guideline testing criteria[b]
**AIDS-defining:**			
Tuberculosis	A15–A19	X	X
Pneumonia, recurrent	J10.0, J11.0, J12–J18 (≥ 2 episodes within 1 year, ≥3 months apart)	X	X
Mycobacteria excluding tuberculosis, disseminated or extrapulmonary	A31.2, A31.8, A31.9	X	X
Pneumocystis carinii pneumonia	B59	X	X
Salmonellasepticemia, recurrent	A02.1	X	X
CMV retinitis or other excluding liver, spleen, glands	B25.0, B25.2, B25.8, B25.9	X	X
HSV bronchitis/pneumonitis	B00.7, B00.8	X	X
Progressive multifocal leukoencephalopathy	A81.2	X	X
Cerebral toxoplasmosis	B58.2	X	X
Cryptosporidiosis, >1 month	A07.2	X	X
Isopsoriasis, > 1 month	A07.3	X	X
Atypical disseminated leishmaniasis	B55	X	X
American trypanosomiasis reactivation (meningoencephalitis or myocarditis)	B56	X	X
Candidiasis (oesophagus or lower respiratory tract)	B37.8, B37.1	X	X
Cryptococcosis, extra-pulmonary	B45 excluding B45.0	X	X
Histoplasmosis, disseminated/extra-pulmonary	B39 excluding B39.0	X	X
Coccidioidomycosis, disseminated/extra-pulmonary	B38	X	X
Cervical cancer	C53	X	X
Non-Hodgkin lymphoma	C82–C85, B21.1 [c], B21.2 [c]	X	X
Kaposi's sarcoma	C46, B21.0*	X	X
**Other than AIDS-defining:**			
Chlamydia	A55–A56	x	x
Syphilis	A51–A53	x	x
Gonorrhoea	A54	x	x
Other sexually transmitted infection (STI)	A57–A64	x	x
Herpes zoster	B02	x	
Hepatitis B and C	B16.0, B16.1, B16.2, B16.9, B17.1, B18.0, B18.1, B18.2	x	x
Invasive pneumococcal disease	A40.3, G00.1, I30.1, M00.1	x	
Candidemia or candida endocarditis	B37.7, B37,6	x	
Visceral leishmaniasis	B55	x	
Hepatitis A	B15.0, B15.9	x	
Community-acquired pneumonia	J10.0, J11.0, J12–J18	x	
Candidiasis excluding genital candidiasis	B37.0, B37.2, B37.4, B37.5, B37.9	x	

Exposure to STI	Z20.2		x
Exposure to HIV	Z20.6		x
Fever on a tropical traveller [b]	B50–B54 (malaria), A97 (dengue)		x
Malignant lymphoma	B21.3 [c], C81	x	
Anal cancer or dysplasia	C20, C21, D01.3, D03.5, D04.5	x	
Cervical dysplasia	D06, N87.2	x	
Primary lung cancer	C34	x	
Mononucleosis-like illness	B27.8, B27.9	x	x
Viral exanthema	B09	x	x
Unexplained fever	R50.9	x	x
Unexplained weight loss	B22.2 [c], R63.4	x	
Unexplained lymphadenopathy	R59, B23.1	x	x
Unexplained chronic renal impairment	N18.9, N19	x	
Leukopenia/thrombopenia > 4 weeks	D70.89, D72.8, D72.9, D69.4, D69.6	x	
Lymphocytic meningitis	A87.2	x	
Oral hairy leucoplakia	K13.3	x	
Guillain-Barré syndrome	G61.0	x	
Mononeuritis	G56–G58, G59.8	x	
Peripheral neuropathy	G61.8, G61.9, G62.9, G64, M79.2	x	
Pregnancy	O, Z34, Z35	x	x

[a] European Centre for Disease Prevention and Control. Public health guidance on HIV, hepatitis B and C testing in the EU/EEA – An integrated approach. Stockholm: ECDC; 2018.
Diagnosis was included here if AIDS-defining or HIV prevalence known or suspected over 0.1%. The diagnoses where testing is indicated if the disease is severe or long-lasting (psoriasis, seborrheic eczema, genital candidiasis, and chronic unexplained diarrhoea) were not included because we could not determine severity or duration from the data. Opioid use was not included as an indicator condition because injection drug use is not usually coded into the diagnosis. Disseminated penicilliosis/talaromycosis is not included due to lack of ICD10 code.

[b] Finnish Institute of Health and Welfare. Brummer-Korvenkontio H, Hiltunen-Back E, Liitsola K, Salminen M. Hiv-testauksen periaatteita: Suositus perusterveydenhuollon toimipisteille ja matalan kynnyksen palvelupisteille. 2010. Briefly: HIV testing is recommended if a person requests the test and as screening in antenatal care, for babies born to women with HIV, and for blood, breast milk, and organ donors. For migrants, screening is currently recommended after arrival for those coming from a high-prevalence (≥1%) country. HIV testing is recommended to be offered by a physician always when an AIDS-indicator disease, sexually transmitted infection, hepatitis B or C, impaired immune function or exposure to HIV is diagnosed or suspected. Additionally, offering HIV testing should be considered by a physician based on an evaluation of individual risk factors including increased risk of HIV exposure (e.g. injection drug use, sex work, men who have sex with men), or symptoms fitting primary HIV infection (mononucleosis-like illness, viral exanthema or fever after traveling to a high-prevalence (>1%) country) or prolonged HIV infection (unexplained fever or lymphadenopathy).

[c] Diagnoses directly connected to HIV (B20–B24) were included only if the diagnosis was made concurrent to HIV diagnosis (i.e., during same inpatient period). Otherwise, the relation to HIV diagnosis was considered unclear and the contacts were excluded from the data.
